# Adaptive Changes and Genetic Mechanisms in Organisms Under Controlled Conditions: A Review

**DOI:** 10.3390/ijms26052130

**Published:** 2025-02-27

**Authors:** Yu-Wei Guo, Yang Liu, Peng-Cheng Huang, Mei Rong, Wei Wei, Yan-Hong Xu, Jian-He Wei

**Affiliations:** 1 Key Laboratory of Bioactive Substances and Resources Utilization of Chinese Herbal Medicine, Ministry of Education & National Engineering Laboratory for Breeding of Endangered Medicinal Materials, Institute of Medicinal Plant Development, Chinese Academy of Medical Sciences and Peking Union Medical College, Beijing 100193, China; rainvy0802@163.com (Y.-W.G.); yliu@implad.ac.cn (Y.L.); huangpengcheng@implad.ac.cn (P.-C.H.); rongmei@implad.ac.cn (M.R.); weiwei@implad.ac.cn (W.W.); 2 Hainan Provincial Key Laboratory of Resources Conservation and Development of Southern Medicine & Key Laboratory of State Administration of Traditional Chinese Medicine for Agarwood Sustainable Utilization, Hainan Branch of the Institute of Medicinal Plant Development, Chinese Academy of Medical Sciences and Peking Union Medical College, Haikou 570311, China

**Keywords:** adaptive changes, controlled conditions, specialized metabolites, classical genetic mechanisms, epigenetic mechanisms

## Abstract

Adaptive changes encompass physiological, morphological, or behavioral modifications occurring in organisms in response to specific environmental conditions. These modifications may become established within a population through natural selection. While adaptive changes can influence individuals or populations over short timeframes, evolution involves the inheritance and accumulation of these changes over extended periods under environmental pressures through natural selection. At present, addressing climate change, emerging infectious diseases, and food security are the main challenges faced by scientists. A comprehensive and profound understanding of the mechanisms of adaptive evolution is of great significance for solving these problems. The genetic basis of these adaptations can be examined through classical genetics, which includes stochastic gene mutations and chromosomal instability, as well as epigenetics, which involves DNA methylation and histone modifications. These mechanisms not only govern the rate and magnitude of adaptive changes but also affect the transmission of adaptive traits to subsequent generations. In the study of adaptive changes under controlled conditions, short-term controlled experiments are commonly utilized in microbial and animal research to investigate long-term evolutionary trends. However, the application of this approach in plant research remains limited. This review systematically compiles the findings on adaptive changes and their genetic foundations in organisms within controlled environments. It aims to provide valuable insights into fundamental evolutionary processes, offering novel theoretical frameworks and research methodologies for future experimental designs, particularly in the field of plant studies.

## 1. Introduction

In natural environments, organisms encounter diverse environmental pressures, including abiotic stressors such as temperature fluctuations, resource limitations, drought, and high salinity, as well as biotic factors like predation and pathogens [1]. These pressures drive adaptive modifications that enhance survival and reproductive success. The duration required for such adaptations varies, depending on the species, environmental conditions, and the nature of the change, ranging from a few generations to millions of years [2]. Several factors influence the rate of evolution, including the intensity of environmental fluctuations [3], survival constraints [4], genetic diversity [5], and genetic drift [6].

Investigating adaptive changes often necessitates controlled cultivation environments to precisely assess the impact of specific environmental variables on organisms. Previous research has identified notable adaptive modifications in various species through multigenerational cultivation under controlled conditions. For instance, in microorganisms, adaptive changes may involve increased resistance to environmental stressors like high temperatures or toxins. In animals, these changes can manifest as alterations in reproductive traits or stress responses. In plants, adaptive changes often include adjustments in leaf morphology or root structure in response to water availability or light conditions. These modifications manifest in physiological, biochemical, morphological, and behavioral traits [7]. Consequently, continuous monitoring of dynamic trait changes through multigenerational cultivation has emerged as a valuable approach for understanding evolutionary mechanisms within a relatively short timespan [8]. This method offers novel opportunities for studying biological evolution [9,10,11].

Research on adaptive modifications under controlled conditions has primarily focused on animals [12,13,14,15] and microorganisms [16,17], while studies on plants remain limited. In plants, biochemical traits, particularly specialized metabolites, are essential for defense against pathogens and herbivores, environmental adaptation, signal transduction, and interspecies interactions [18,19,20,21]. These metabolites are crucial for plant survival and reproduction, and have significant applications in agriculture and medicine [22,23].

Currently, a substantial knowledge gap exists regarding how specialized metabolites change under controlled conditions, the mechanisms governing these changes, and potential regulatory pathways. Multigenerational plant cultivation under controlled conditions, similar to research in microorganisms (e.g., *Saccharomyces cerevisiae*) and animals (e.g., *Drosophila melanogaster*), could facilitate a deeper understanding of the evolutionary mechanisms regulating specialized metabolite production. *Saccharomyces cerevisiae* is widely used as a model organism in evolutionary biology due to its short generation time and well-characterized metabolic pathways. *Drosophila melanogaster*, selected for its extensively characterized genetic background and the ease with which multigenerational populations can be maintained, provides an invaluable model for studying genetic adaptation. In contrast, *Arabidopsis thaliana* has been selected for its genetic tractability and significance in plant biology. However, further research is required to gain a deeper understanding of its specialized metabolite production under controlled conditions (figure in Section 3.1.1). A comprehensive summary of adaptive changes in organisms under controlled environments would provide a foundation for elucidating biodiversity and adaptability, advancing knowledge of organism–environment interactions, and contributing to agricultural and medical advancements while uncovering evolutionary pressures and selection mechanisms.

## 2. Adaptive Changes in Organisms Under Controlled Conditions

### 2.1. Adaptive Changes in Microorganisms

In microbiology, multigenerational cultivation of fungi under controlled conditions results in substantial modifications in morphological traits, physiological characteristics, reproductive attributes, and biochemical indicators.

#### 2.1.1. Changes in Morphological, Physiological, and Reproductive Traits

**Figure 1:**
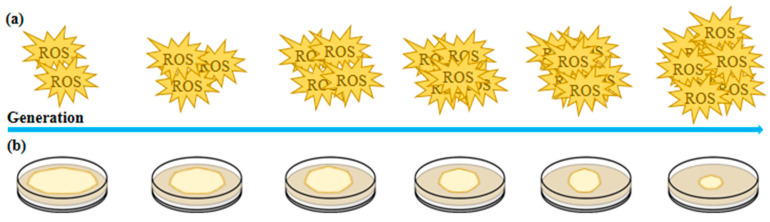
Trends in the adaptive changes in microbial traits under controlled conditions. (**a**) Multigenerational cultivation gradually increases reactive oxygen species (ROS) levels in microorganisms. (**b**) Multigenerational cultivation leads to a progressive decline in microbial colony size and biomass.

After 12 consecutive subcultures of fungi, significant signs of strain degradation were observed, including reduced antioxidant enzyme activity and increased reactive oxygen species (ROS) levels (Figure 1). With each successive passage, additional indicators of cellular stress emerged, such as a decline in nuclear number, decreased mitochondrial membrane potential, and alterations in mitochondrial morphology. These findings suggest that ROS accumulation, combined with repeated tissue isolation, exacerbates oxidative damage in inherited strains, ultimately contributing to cellular senescence and degeneration in *Volvariella volvacea* strains [24,25]. Continuous subculturing of *Volvariella volvacea* strains over 20 months, with subcultures performed every three days, resulted in a progressive decline in growth rate, mycelial biomass, fruiting body count, and bioefficiency as the number of subcultures increased. Simultaneously, the production cycle and primordium formation time were progressively extended. Strains subcultured for 13 to 20 months (S13–S20) failed to produce fruiting bodies during cultivation experiments. Additionally, ROS content measurements and enzyme activity analyses indicated a decline in lignocellulase activity and excessive ROS accumulation, both of which were associated with subculture-induced degeneration. Reverse transcription polymerase chain reaction (RT-PCR) analysis of gene expression related to lignocellulase and antioxidant enzymes in the subcultured strains demonstrated consistency with the observed enzymatic activity changes [26].

#### 2.1.2. Changes in Biochemical Indicators

In microbiology, biochemical indicators undergo significant changes following multigenerational cultivation under controlled conditions, which typically involve maintaining a constant temperature, pH, light exposure, and nutrient supply. These conditions ensure that the organisms are exposed to minimal external variability, allowing for the observation of genetic and phenotypic changes over multiple generations. A notable trend is the decline in specialized metabolite content with an increasing number of subcultures. However, certain experiments have demonstrated that mimicking environmental stimuli can restore strains’ productivity, allowing for the renewed production of specialized metabolites. The loss of productivity for compounds such as paclitaxel, camptothecin, and cordycepin during laboratory storage or successive subculturing has been linked to the absence of host-related stimuli. Chen et al., (2017) conducted 10 consecutive subcultures of a wild-type Cordyceps strain, resulting in strain degeneration. By hybridizing four monospore isolates derived from the degenerated strain, fruiting body production was restored to levels comparable with the wild-type strain. The revitalized strain not only exhibited well-developed fruiting bodies but also accumulated higher concentrations of cordycepin and adenosine than both the original wild-type and degenerated strains. These newly acquired traits remained stable after four consecutive transfers [27]. Similarly, nutrient optimization in *Penicillium chrysogenum* was shown to enhance camptothecin production by 1.8 times. However, after 8 months of storage at 4 °C, camptothecin productivity declined by 40% compared with the initial culture. The application of a dichloromethane extract from *Cliona* sp. was found to fully restore the ability of *P. chrysogenum* to produce camptothecin [28]. Successive subcultures of *Aspergillus terreus* have been reported to cause a substantial decline in paclitaxel production. By the 10th subculture, the paclitaxel yield (78.2 μg/L) was reduced to one-fourth of that observed in the original culture (268 μg/L). Notably, supplementation with the plant microbiome fully restored both paclitaxel biosynthesis and cellular acetyl-CoA levels, indicating that the introduction of the plant microbiome can reactivate the molecular mechanisms responsible for paclitaxel biosynthesis [29]. After continuous subculture, a reduction in the production of specialized metabolites, such as cordycepin, has been noted. During subculturing on solid media, filamentous fungal colonies frequently undergo degeneration, which is characterized by the loss of conidia, pigment deposition, and a marked decline or a complete loss of specialized metabolite production [30]. Wellham et al., (2021) reported that repeated subculturing of *Cordyceps militaris* under laboratory conditions led to strain degeneration, evidenced by a reduction in cordycepin production [31]. Similarly, Chen et al. (2017) also observed that multigenerational cultivation of *Cordyceps* resulted in decreased synthesis of specialized metabolites, including cordycepin [27].

### 2.2. Adaptive Changes in Animals

In animal studies, multiple species have demonstrated notable adaptive modifications following multigenerational cultivation under controlled environments, such as stable temperature, humidity, and light conditions, along with a controlled food supply and selective pressure (e.g., temperature stress, limited resources). These modifications encompass changes in reproductive and fertility-related phenotypes, physiological and lifespan-associated traits, behavioral and life history characteristics, as well as morphological traits [32]. A comprehensive summary of all the observed phenotypic changes and the extent of transgenerational cultivation in the studied animal species is presented in Table 1.

#### 2.2.1. Changes in Reproductive and Fertility-Related Phenotypes

Reproductive and fertility-related phenotypic modifications have been observed under controlled cultivation conditions across multiple generations. For example, rapid phenotypic adaptations have been documented in traits such as mean fecundity (number of eggs per female per day), egg size, starvation tolerance (measured by time to death due to starvation), and recovery time following chill coma. The extent of these adaptations was considerable, with at least 165 independent genomic regions identified as being under selection during the experiment. Allele frequencies at more than 60% of variant sites across the genome were partially influenced by selection, indicating significant genetic adaptation to the controlled environment [33]. A multigenerational laboratory study using females from two populations of *Heterandria formosa* investigated transgenerational plasticity in reproductive traits in response to social density variations and their impact on maternal fitness. The findings indicated that increased social density resulted in a lower reproductive rate and larger offspring size in females from both populations during the first and second generations [34]. Additionally, parental exposure to a common pesticide was shown to induce intra-, inter-, and transgenerational responses in life history traits such as fecundity, longevity, and lifetime reproductive success, as demonstrated in studies using *Callosobruchus maculatus* as an insect model. The results revealed sex-specific and hormetic intergenerational and transgenerational effects on longevity and reproductive success, with both maternal and paternal contributions to these effects [35]. Research on *Daphnia pulicaria* has shown that the first generation exhibited reduced fitness traits, including delayed maturation, lower reproductive output, and increased clutch interval when reared on cyanobacteria compared with high-quality food. However, maternal stress had no significant impact on the offspring’s fitness traits, as the second generation displayed similar mean trait values regardless of maternal diet [36]. In *Aurelia coerulea* polyps, transgenerational effects on traits such as budding reproduction and strobilation were examined over 10 asexual generations. The results indicated a 32.82% decline in the average budding reproduction rate across asexual generations within the experimental period [37]. Studies on wild-derived mice after eight generations under laboratory conditions have demonstrated rapid adaptation to reintroduced sociality and promiscuity within two generations. Males from the promiscuous lineage exhibited reduced viability but gained a considerable advantage in mate attraction through increased expression of major urinary protein (MUP) pheromones, which were inherited transgenerationally. This enhanced MUP expression was associated with reduced DNA methylation in the promoter region, leading to heightened female attraction [45].

#### 2.2.2. Changes in Physiological and Lifespan-Related Phenotypes

Physiological and lifespan-related phenotypic modifications have been observed following an extended diapause in *Caenorhabditis elegans*. Initially, post-diapause individuals exhibit reduced reproductive success and greater interindividual variation. However, the F3 progeny of these individuals demonstrate enhanced starvation resistance and increased lifespan, suggesting potentially adaptive transgenerational effects [38]. Experimental evolution studies on an insect model system have investigated the role of sexual selection history in shaping transgenerational plasticity in response to rapid environmental changes, such as pesticide exposure. In a study with *Callosobruchus maculatus*, it was demonstrated that deltamethrin, a neurotoxic pyrethroid pesticide (applied at 2 g/L for 24 h), induces both transgenerational plasticity and hormetic responses to sublethal exposure. The results revealed an extension of lifespan in F2 descendants, alongside hormetic responses modulated by the intensity of sexual selection history. After varying the intensity of sexual selection over more than 80 generations before pesticide exposure, the findings indicated that sexual selection history constrained adaptation to rapid environmental change. Additionally, inter- and transgenerational effects of pesticide exposure were observed, leading to increased fitness and longevity in subsequent generations [39]. The transgenerational and within-generation plasticity of anti-predator traits have been examined in the freshwater snail *Physa acuta*. Across two generations, most morphological traits, including shell size and crushing resistance, exhibited transgenerational plasticity, with the offspring of predator-exposed parents being larger and more resistant to crushing. However, shell shape exhibited plasticity only within a single generation [40]. In the freshwater crustacean *Daphnia lumholtzi*, exposure to chemical cues from fish predators has been shown to induce the development of elongated head and tail spines. In the F0 generation, these defensive traits exhibited a prolonged lag phase, emerging only by the third instar. However, in the F1 and F2 generations, this lag phase was reduced, with defensive traits appearing immediately upon birth, indicating a shift in phenotypic expression across generations [41].

#### 2.2.3. Changes in Behavioral and Life History Traits

Behavioral and life history trait modifications have been observed in response to transgenerational exposure to ultraviolet radiation (UVR). A reciprocal split-brood experiment conducted over 150 generations demonstrated that transgenerational responses to UVR varied depending on ancestral exposure. Descendants from unexposed ancestor lineages exhibited enhanced phototactic avoidance and delayed reproduction upon initial exposure to ultraviolet radiation (UVR), whereas descendants originating from UVR-exposed populations demonstrated reduced stress responses and accelerated larval development under low-UVR conditions. These patterns reveal a fundamental mechanism by which ancestral environmental conditions shape adaptive plasticity: Populations with no prior UVR exposure prioritize immediate survival through rapid phenotypic adjustments, while those adapted to historical UVR exposure maintain energy-conserving behaviors as the threat diminishes. This divergence in phenotypic strategies across generations highlights the dynamic interplay between evolutionary history and the environmental context in mediating organismal responses to fluctuating stressors [42]. Transgenerational phenotypic responses of pea aphids to ladybird predators have been examined over 27 generations. The frequency of winged aphids initially increased rapidly and remained stable for 25 generations. After predator removal, the winged phenotype persisted for one generation before entering a refractory phase, during which its frequency dropped below control levels for at least two generations before recovering. With prolonged predator exposure, the persistence of the winged phenotype declined, and the refractory phase was extended. Additionally, aphids exposed to predators for 22 generations exhibited a reduced plastic response, which conferred a fitness advantage in the presence of predators [43].

#### 2.2.4. Changes in Morphological Traits

Examples of morphological trait modifications include a study by Mark Smithson, which investigated the stability of an adaptive phenotype (shell shape) and DNA methylation across generations in a clonally reproducing freshwater snail (*Potamopyrgus antipodarum*). Three generations of snails, originally adapted to river currents, were reared in a laboratory environment without a current. The results indicated that the habitat-specific adaptive shell shape remained relatively stable across three generations but exhibited a slight shift in the second and third generations toward a no-current lake phenotype [44].

### 2.3. Adaptive Changes in Plants

In plant studies, multigenerational cultivation under controlled conditions results in notable modifications in morphological traits and biochemical indicators.

#### 2.3.1. Changes in Plant Morphology

**Figure 2:**
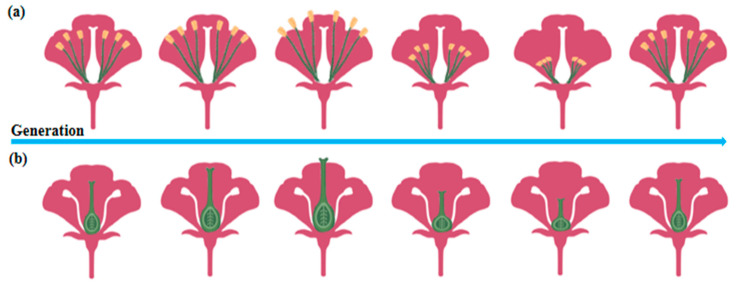
Trends in plant trait adaptation under controlled conditions. (**a**) Multigenerational cultivation initially increases stamen length, followed by a subsequent decrease in plants. (**b**) Multigenerational cultivation causes an initial rise in pistil length, followed by a gradual decline in plants.

In plant biology, multigenerational cultivation under controlled conditions results in significant morphological modifications. After 11 consecutive generations of co-cultivation with pollinators, bumblebee-pollinated plant species exhibited a notable increase in plant height. Additionally, pollination efficiency, measured by the number of seeds per fruit, showed a steady improvement throughout the experimental period [46]. Real-time evolutionary tracking of plant traits over six generations using rapidly cycling Brassica plants revealed substantial changes in reproductive structures. The lengths of both the pistil and long stamens initially shortened before exhibiting a subsequent elongation trend (Figure 2), highlighting dynamic trait adjustments influenced by the presence or absence of pollinators [47]. Similarly, somatic embryos serve as optimal material for woody plant propagation, germplasm conservation, and genetic transformation. A comparison of grape proembryonic masses (PEMs; early-stage plant tissues undergoing embryogenesis during in vitro culture) that underwent continuous tissue culture for 10 years under controlled environmental conditions (e.g., a constant temperature, light cycles, and nutrient supply) with newly induced PEMs revealed a significant reduction in somatic embryo induction rates in long-term PEMs, with an abnormal embryo ratio reaching 92.2%. Additionally, the classical cellular cluster structure nearly disappeared in repeatedly subcultured PEMs, along with an increase in the space between adjacent cells [48]. Exposure of 14 Arabidopsis thaliana genotypes to 10 distinct controlled environmental stresses over four consecutive generations led to pronounced phenotypic alterations in most genotypes. Under stable nutrient stress, aboveground biomass consistently increased with each successive generation. However, fruit yield displayed variable responses, showing a marked increase under jasmonic acid treatment but a significant decrease under high cadmium stress, which was also associated with reduced plant stature [49]. To assess the extent of stress-induced transgenerational adaptation, *Arabidopsis thaliana* (including Col-0) was subjected to two distinct hyperosmotic conditions for more than five generations. The direct progeny (P1) of stressed plants from generations G2 to G5 exhibited higher germination and survival rates, along with more vigorous vegetative growth when cultivated in high-salinity (150 mM) media [50].

#### 2.3.2. Changes in Biochemical Parameters

Multigenerational cultivation of plants has resulted in significant alterations in biochemical indicators. After 11 consecutive generations of co-cultivation with pollinators, notable shifts in floral volatile profiles were observed. Nearly half of the plants pollinated by flies had ceased anisaldehyde emission by the 11th generation. In contrast, plants pollinated by bumblebees exhibited a substantial increase in indole and glutaraldehyde content, along with a decline in anisaldehyde levels, particularly in response to aphid herbivory [46]. Real-time evolutionary tracking of plant traits over six generations using rapidly cycling *Brassica* plants revealed a significant rise in the emission of three aromatic floral volatiles—benzaldehyde, benzonitrile, and p-anisaldehyde—in plants exposed to bee-pollinated conditions. These findings emphasize the role of pollinators in shaping floral volatile composition across successive generations [47]. Plant in vitro culture, a crucial technique in plant research, is significantly affected by multigenerational cultivation. In grape proembryonic masses (PEMs), repeated subculturing led to a considerable decline in cell wall pectin content [48].

## 3. Genetic Mechanisms and Their Role in Adaptive Changes

Genetic mechanisms, encompassing both classical and epigenetic processes, play a central role in driving adaptive changes across diverse organisms. The interplay between classical and epigenetic mechanisms is particularly evident in their roles across different taxa. In microorganisms, rapid mutation rates and horizontal gene transfer drive the evolution of antibiotic resistance and metabolic versatility. In animals, genetic and epigenetic changes underpin adaptations to climatic shifts, dietary changes, and pathogen pressures. In plants, epigenetic modifications enable responses to abiotic stresses, such as drought or salinity, while genetic diversity supports long-term evolutionary resilience. Together, these mechanisms form an integrated framework that shapes adaptive changes at both the organismal and population levels, highlighting their fundamental importance in evolutionary biology.

### 3.1. Genetic Mechanisms

#### 3.1.1. Classical Genetic Mechanisms

Classical genetic mechanisms encompass a range of processes, including random gene mutations, chromosomal instability, and genetic variations. Random genetic mutations, which occur spontaneously at low frequencies, represent a fundamental driver of evolutionary change. Chromosomal instability has also been recognized as a significant contributor to the loss of biosynthetic genes, influencing genomic architecture and functional diversity. Furthermore, genetic variation serves as a critical determinant in shaping the structural diversity of specialized metabolites across populations, thereby underpinning adaptive potential and ecological interactions.

**Figure 3:**
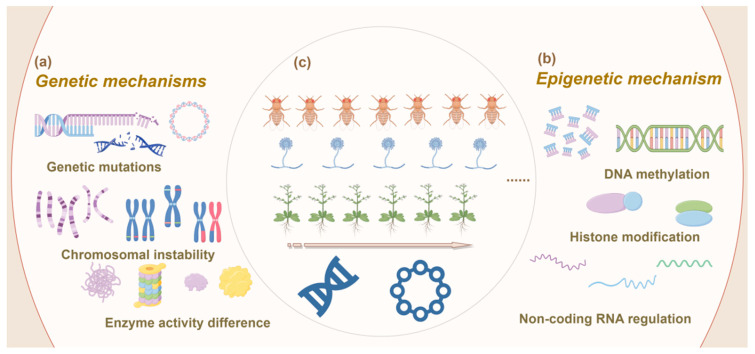
Schematic representation of genetic mechanisms driving adaptive changes in organisms under controlled conditions. (**a**) Genetic mechanisms: This panel illustrates the key genetic processes involved in adaptive changes, including gene mutations, which introduce new genetic variations; chromosomal instability, which involves structural alterations of chromosomes, such as inversions or translocations; and enzyme activity differences, which influence metabolic pathways and can drive phenotypic changes. (**b**) Epigenetic mechanisms: This panel depicts various epigenetic processes that regulate gene expression without altering the underlying DNA sequence. These include DNA methylation, which typically suppresses gene expression; histone modifications, such as acetylation or methylation, which alter chromatin structure and affect gene accessibility; and non-coding RNA regulation, which involves RNA molecules that can modulate gene expression post-transcriptionally. (**c**) Multigenerational cultivation across different organisms (microorganisms, animals and plants): This panel represents the application of controlled multigenerational cultivation to study adaptive changes in different organisms. At the top are animals, represented by *Drosophila melanogaster* (fruit flies), known for their genetic tractability and rapid generation time; in the middle are microorganisms, represented by fungi, particularly species like *Saccharomyces cerevisiae*, which are commonly used in evolutionary studies due to their short life cycle and high mutation rates; at the bottom are plants, represented by *Arabidopsis thaliana*, a model organism in plant biology due to its well-characterized genetics and significance in plant research. The figure highlights how different organisms are used to explore the genetic and epigenetic mechanisms that drive adaptation under controlled experimental conditions.

Dynamic genetic modifications are fundamental drivers of adaptive variations in organisms. Processes such as random genetic mutations, gene and whole-genome duplications, gene neofunctionalization, chromosomal rearrangements, horizontal gene transfer, and chromatin remodeling contribute to evolutionary changes [51,52,53] (Figure 3a). As genetic variations emerge, adaptive traits in organisms are modified, leading to adjustments in specialized metabolic pathways that influence both the types and quantities of metabolites produced. In plants, most specialized metabolic pathways are likely established through successive duplications and neofunctionalization of endogenous genes, whereas in fungi, some metabolic pathway genes may also evolve through horizontal gene transfer [54,55]. This complex interplay provides a strong genetic foundation for biological adaptation.

#### 3.1.2. Epigenetic Mechanisms

In 1958, Nanney introduced the concept of “epigenetics” to differentiate whether observed variations in cellular characteristics resulted from changes in the “original genetic material” or other cellular components. This concept underscored the dependence of the epigenetic system on the genetic system and its significance during development [56]. Epigenetics refers to heritable modifications in gene expression that occur without alterations in the DNA sequence, primarily mediated by mechanisms such as DNA methylation, histone modifications, and regulation by non-coding RNAs, including miRNAs (Figure 3b). These epigenetic changes often arise in response to environmental stimuli and interact to modulate gene expression, ultimately shaping cellular phenotypes [57].

Epigenetic mechanisms comprise three major regulatory modalities: DNA methylation, histone modifications, and non-coding RNA-mediated regulation. Among these, DNA methylation serves as a fundamental epigenetic mark, playing a pivotal role in gene silencing, genomic imprinting, and chromatin remodeling. Histone modifications, including acetylation, methylation, and phosphorylation, constitute another critical layer of epigenetic regulation by dynamically altering the chromatin structure and modulating gene expression. Additionally, non-coding RNAs, such as microRNAs and long non-coding RNAs, have emerged as essential regulators of epigenetic processes, fine-tuning gene expression through transcriptional and post-transcriptional mechanisms. Collectively, these interconnected mechanisms form a sophisticated regulatory network that governs cellular function and phenotypic plasticity. Epigenetic modifications play a crucial role in the intricate mechanisms that facilitate adaptive changes in organisms.

### 3.2. The Role of Genetic Mechanisms in Adaptation

#### 3.2.1. Adaptation in Microorganisms

After 12 successive subcultures, *Volvariella volvacea* strains exhibited pronounced degenerative phenotypes, marked by a significant decline in antioxidant enzyme activity and a concomitant increase in reactive oxygen species (ROS) accumulation (Figure 1). These degenerative changes are mechanistically linked to oxidative stress-induced chromosomal instability, evidenced by increased chromosomal fragmentation and aneuploidy, and genetic variation, characterized by the accumulation of point mutations and structural rearrangements. These alterations progressively disrupt genomic integrity and gene regulatory networks during repeated subculturing, ultimately driving cellular dysfunction and senescence in fungal strains [24,25].

Continuous subculturing of *Volvariella volvacea* strains over a period of 20 months resulted in a progressive decline in growth rate, mycelial biomass, and fruiting body production, along with prolonged production cycles and delayed primordium formation. Strains subcultured for 13 to 20 months (S13–S20) completely lost the ability to form fruiting bodies. The mechanistic basis for this degeneration involves two interconnected processes: Genetic variation and non-coding RNA dysregulation. Genetic variation, driven by the accumulation of point mutations and structural rearrangements, led to the downregulation of several key genes, including *LAC-1*, *LAC-4*, *EG-B*, *CBH*, *BGL*, *Xyl*, *Mn-SOD*, *CAT-1*, *CAT-2*, *GPX*, and *GR*. Among these, *LAC-1* and *LAC-4* exhibited the most significant decline in expression, decreasing by 77.36% and 72.67%, respectively, at S20 compared with S0. This reduction directly impaired lignin degradation and fruiting body morphogenesis. Simultaneously, excessive reactive oxygen species (ROS) accumulation disrupted non-coding RNA regulatory networks, which further suppressed the expression of genes involved in lignocellulase production and antioxidant enzyme activity. This dual mechanism of genetic instability and epigenetic dysregulation progressively compromised cellular function, nutrient metabolism, and stress response, ultimately leading to the loss of fruiting body formation and reduced bioefficiency [26].

In fungi, high mutation rates are often associated with transposable element (TE) activity or alterations in chromosomal ploidy [58,59]. Although mutations originate from low frequencies, their prevalence can increase due to positive selection, where novel phenotypes providing a selective advantage, gradually replacing the wild-type [60]. High-frequency mutations resulting from transposable element activity have been documented in multiple cases of fungal degeneration during biotechnological applications. For example, Zhong et al., (2018) reported a decline in hydrolase productivity [61], while Christensen et al., (1995) observed reduced penicillin production in *P. chrysogenum*, both illustrating this phenomenon [62].

Research indicates that the degeneration of penicillin production capability is linked to high-producing strains of penicillin-producing fungi, which contain an increased copy number of biosynthetic gene clusters arranged in tandem repeats [63,64,65]. These amplifications are believed to result from misaligned chromatids and recombination events occurring within recombinogenic regions. The same mechanisms may also lead to the deletion of genes involved in penicillin biosynthesis [65]. Similarly, in fungi, chromosomal instability may drive the expansion or contraction of gene clusters through mechanisms like non-homologous end joining (NHEJ) or homologous recombination, thereby modulating metabolic pathways in response to selective pressures [66]. The presence of multiple copies of penicillin biosynthetic cluster genes, especially during extended cultivation under conditions that are unfavorable for penicillin production, may impose a metabolic burden on fungal cells. Under such conditions, cells with fewer or no copies of penicillin cluster genes may experience a selective advantage [66]. These findings suggest that the regulation of gene copy number and genetic architecture, influenced by both chromosomal instability and environmental factors, plays a key role in maintaining the long-term stability and productivity of industrial microbial strains.

It has been recognized as a key factor contributing to the degradation of *Cordyceps militaris* strains. Variations in gene expression levels and enzymatic activities among different genotypes lead to significant alterations in specialized metabolite profiles, highlighting the genetic basis of strain degeneration [67]. In citric acid-producing strains, degeneration is characterized by a substantial downregulation of the genes involved in its biosynthesis, including pathways associated with the tricarboxylic acid (TCA) cycle, glycolysis, starch and sucrose metabolism, and pyruvate metabolism. These disruptions illustrate the molecular mechanisms underlying strain degradation [68].

The application of the epigenome-targeting CRISPR/dCas9 system to regulate specialized metabolism genes in *Aspergillus niger* marked a significant advancement in this field. By fusing HosA-dCas9 with the pigment gene *fwnA*, it was demonstrated that the histone deacetylase HosA activated *fwnA* expression, leading to an acceleration of melanin synthesis. This approach underscores the potential of epigenetic manipulation in enhancing metabolic pathways in fungi, providing opportunities for targeted improvements in specialized metabolite production [69]. In *Penicillium* sp. KM18029, an endophytic fungus isolated from *Aconitum brachypodum* Diels, meroterpenoid biosynthesis was observed. Li et al., (2022) utilized histone deacetylase inhibitors as epigenetic modifiers, leading to the isolation of four novel compounds from *Penicillium* sp. KM18029. This study demonstrated that epigenetic modifications can serve as an effective strategy for activating terpenoid biosynthetic gene clusters in *Penicillium* sp. [70].

#### 3.2.2. Adaptation in Animals

In animal research, Pierron et al., (2023) utilized copy number variations (CNVs) to investigate the potential role of gene mutations in transgenerational processes. While CNVs were relatively rare in the F1 generation, their frequency significantly increased in the sperm of the F3 generation, suggesting that environmentally induced epigenetic modifications may contribute to genomic instability and facilitate the emergence of CNV mutations in offspring. Cadmium (Cd) exposure has been shown to induce epigenetic effects across four generations in zebrafish, primarily through DNA methylation changes in the estrogen receptor alpha gene (*esr1*). These methylation modifications were sex-dependent and inherited transgenerationally. Cd-induced methylation changes were linked to single nucleotide polymorphisms (SNPs), which facilitated genetic selection and influenced gene transcription. While Cd exposure contributed to transgenerational disease susceptibility, it also promoted rapid transgenerational adaptation to Cd. These findings provide insights into the mechanisms of rapid adaptation and highlight the crucial role of genetic diversity in enhancing species’ resilience to global environmental changes [71]. Exposure to the pesticide DDT significantly influenced trait expression in male rats across multiple generations, primarily through the transgenerational inheritance of differential DNA methylation regions (DMRs) in the sperm. From the F3 generation onward, DDT exposure induced specific DMR alterations during sperm development, affecting key stages such as embryonic prospermatogonia, juvenile spermatogonia, adult spermatocytes, round spermatids, and caudal epididymal sperm. Notably, DMR changes originating during the spermatogonia stage in the testes were inherited by offspring through epigenetic mechanisms, leading to alterations in gene expression patterns across generations [72].

Chromosomal aberrations, including inversions and translocations, are prevalent among eukaryotic organisms and have been widely implicated in adaptive evolution. A prominent example is the role of heterozygous inversions in *Drosophila melanogaster*, a model organism extensively utilized in cytogenetic research. These inversions have been demonstrated to generate genetic variation that is subject to rapid selection under environmental stress. Notably, the In(3R)Payne inversion in *D. melanogaster* has been shown to play a pivotal role in shaping adaptive clines for traits such as body size and thermal tolerance. For instance, In(3R)Payne has been strongly associated with latitudinal variation in body size, accounting for over 70% of clinal size differences observed along the east coast of Australia and in parallel South American clines. This inversion likely facilitates adaptation by suppressing recombination, thereby preserving co-adapted gene complexes that enhance fitness under specific environmental conditions, such as colder climates, where larger body size confers a selective advantage. Furthermore, In(3R)Payne has been linked to thermal resistance, potentially mediated by genes within the inversion, such as hsr-omega, which exhibit pronounced clinal patterns. These findings underscore the significance of inversions in promoting rapid adaptation by maintaining adaptive allele combinations and responding to environmental gradients, such as temperature and latitude [73].

#### 3.2.3. Adaptation in Plants

In plant biology, a study by Lu et al., (2021) investigated 22 generations of *Arabidopsis thaliana* exposed to heat stress. The research demonstrated that prolonged exposure of mutation accumulation (MA) lines and MA populations to extreme heat and moderate warming resulted in a significant increase in the mutation rate of single nucleotide variants (SNVs) and small insertions and deletions [11]. These findings highlight that environmental changes can accelerate the accumulation of mutations throughout the genome, altering the spectrum of novel mutations within an organism. This process may play a crucial role in long-term evolutionary adaptation, enabling species to better withstand shifting environmental conditions [74,75,76,77,78,79].

The diversity of specialized metabolites, driven by genetic variation, plays a critical role in enhancing organismal adaptability. Genome-wide gene expression analyses conducted across multiple generations of *Arabidopsis thaliana* exposed to control and ten specific environmental treatments (jasmonic acid addition (JA), the control for JA, nutrient addition, nutrient depletion, leaf removal, drought, low cadmium (50 μmol/L CdCl_2_), high cadmium (1500 μmol/L CdCl_2_), low salinity (50 mM NaCl), and high salinity (150 mM NaCl)) have demonstrated that such treatments consistently induce heritable phenotypic and gene expression changes. These findings challenge the conventional view of the environment solely as a selective force for genetic variation, instead highlighting its active role in generating genetic diversity [49]. Genetic evolution is closely linked to adaptability, enabling organisms to modify their genotypes in response to environmental challenges. This process involves the regulation of secondary metabolic pathways, allowing the synthesis of specialized metabolites tailored to specific environmental conditions. Studies have shown that nearly all genetic changes within experimental populations are unique, indicating that evolutionary trajectories are highly divergent. Even genetically similar replicates derived from the same ancestral population can exhibit distinct evolutionary paths under controlled and consistent selection pressures, underscoring the complexity of evolutionary processes [80].

In plant research, a study investigating the transgenerational effects of salt stress on *Arabidopsis thaliana* identified substantial changes in DNA methylation levels over 10 generations under both normal and salt stress conditions. A comparison between two genotypes, namely wild-type and ros1 mutants, revealed that ros1 mutants exhibited more than twice the number of differentially methylated cytosines (DMCs) and a higher CG epimutation rate in the 10th generation compared with the wild-type. In wild-type plants, about 50% of transgenerational DMCs showed increased methylation, whereas this proportion was significantly higher in ros1 mutants. These results indicate that active DNA demethylation plays a critical role in regulating transgenerational increases in DNA methylation, thereby contributing to epigenomic stability [81]. Exposure of *Arabidopsis* plants to two distinct hyperosmotic conditions over five generations identified specific genomic regions susceptible to DNA (de)methylation in response to hyperosmotic stress. These stress-induced epigenetic modifications were linked to conditionally heritable adaptive phenotypic responses, suggesting that hyperosmotic stress can drive epigenetic changes that enhance a plant’s ability to adapt to environmental stressors across generations [50].

Recent studies have emphasized the regulatory roles of non-coding RNAs (ncRNAs) in the biosynthesis of specialized metabolites in plants. miRNAs, lncRNAs, and circRNAs have been shown to interact with coding genes, influencing their expression and subsequently affecting metabolic pathways. These ncRNAs, which do not encode proteins, constitute a substantial portion of the plant transcriptome and play essential roles in modulating the biosynthesis of specialized metabolites by regulating key synthesis-related genes. This expanding body of research highlights the significance of ncRNAs in the intricate regulatory networks governing plant metabolism [82]. Although often examined separately, DNA methylation, histone modifications, and transcription factor activity can integrate to regulate gene expression. Under various stress conditions, significant variations in the expression of genes associated with DNA methylation and histone acetylation were detected in tea leaves. This study demonstrated that both DNA methylation and histone modifications contribute to the regulation of specialized metabolism in tea. Specifically, the transcription of DNA methyltransferase, DNA demethylase, histone acetyltransferase (HAT), and histone deacetylase (HDAC) genes exhibited notable alterations under stress. For instance, the inhibition of HDACs (e.g., with trichostatin A treatment) reduces histone deacetylation, leading to elevated histone acetylation levels at the promoters of target genes, such as *CsTSB2*, *CsLOX1,* and *CsNES.* These genes are crucial for the synthesis of stress-responsive specialized metabolites, including indole and (E)-nerolidol. These findings suggest that epigenetic mechanisms, including DNA methylation and histone modification, play a pivotal role in the metabolic responses of tea plants to environmental stressors [83]. Changes in DNA methylation levels have been shown to suppress carotenoid and anthocyanin production, with modifications in aroma components observed following 5-azacytidine treatment. DNA hypomethylation led to the upregulation of genes involved in photosynthesis, flavonoid biosynthesis, and hormone signaling pathways. However, this increase in gene expression was accompanied by a corresponding decline in protein levels. These findings highlight the crucial role of DNA methylation in regulating key metabolic pathways, impacting both pigment production and specialized metabolism in plants [84]. Although these findings do not directly pertain to the regulation of specialized metabolism genes, it is reasonable to hypothesize that similar epigenetic mechanisms may govern gene expression in these contexts [85].

## 4. Discussion

### 4.1. Environmental Conditions and Adaptive Changes

**Figure 4:**
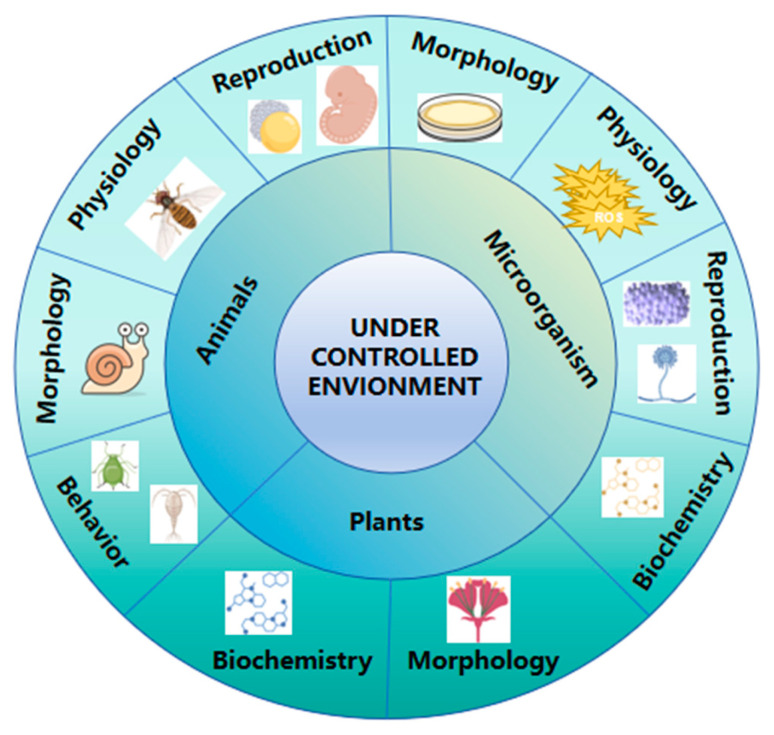
Types of adaptive traits exhibited by different organisms under controlled conditions. In animals, adaptive changes include reproductive, physiological, morphological, and behavioral traits. In microorganisms, variations occur in morphological, physiological, reproductive, and biochemical traits. In plants, adaptations involve morphological and biochemical traits.

The type and intensity of environmental conditions play a pivotal role in determining the direction and magnitude of adaptive changes. For instance, microorganisms subjected to nutrient-limited environments often exhibit rapid morphological and physiological adaptations, such as biofilm formation or metabolic reprogramming, to enhance survival. In contrast, animals exposed to controlled stressors like temperature fluctuations or dietary restrictions may develop adaptive changes in reproductive strategies, lifespan, or behavior (Figure 4). These adaptations have been extensively studied, with well-documented examples such as the rapid evolution of antibiotic resistance in bacteria and the phenotypic plasticity of reproductive traits in rodents under dietary stress.

In plants, however, research on adaptive changes under controlled conditions remains relatively limited, particularly in comparison with the breadth of studies on microorganisms and animals. While some studies have explored morphological and biochemical adjustments, such as changes in stamen and pistil length or stress-induced metabolite accumulation in response to abiotic stresses like drought or salinity, fewer investigations have focused on reproductive traits. For example, the role of seed-related traits—such as seed size, germination rate, and dormancy—in plant adaptations to controlled environments remains underexplored. Given the critical importance of seeds in plant survival and dispersal, future research could leverage insights from microbial and animal studies to investigate how environmental pressures shape seed-related adaptations. By examining these traits, researchers may uncover novel mechanisms of plant adaptation and provide a more comprehensive understanding of evolutionary processes across taxa.

### 4.2. Mechanisms Underlying Adaptive Changes

The diversity of adaptive changes is underpinned by a combination of classical genetic and epigenetic mechanisms. In microorganisms, stochastic gene mutations and chromosomal instability are primary drivers of rapid adaptation, enabling swift responses to environmental challenges. For instance, fungi subjected to successive subculturing often exhibit significant degenerative changes, such as reduced fruiting body production and metabolic activity, which have been linked to chromosomal instability and genetic variation. In animals, adaptive changes in reproductive and physiological traits are often mediated by genetic variation and epigenetic modifications, such as DNA methylation and histone modifications, which fine-tune gene expression in response to environmental cues. A notable example is the role of DNA methylation in regulating stress-responsive genes in mammals exposed to fluctuating temperatures.

In plants, current research has primarily focused on epigenetic mechanisms, with DNA methylation and non-coding RNA regulation being the most extensively studied. For example, DNA methylation has been demonstrated to play a critical role in mediating stress responses, such as drought tolerance in *Arabidopsis thaliana*, by modulating the expression of stress-related genes. Similarly, non-coding RNAs, including microRNAs and long non-coding RNAs, have been implicated in regulating developmental plasticity and stress adaptation in plants. However, chromosomal instability, a well-documented driver of adaptation in microorganisms and animals, has not been widely reported or summarized as a major factor in plant adaptation. This discrepancy may reflect a true biological difference or a gap in current research. Exploring the potential role of chromosomal instability in plant adaptations, particularly under extreme environmental stressors, could uncover novel genetic and epigenetic mechanisms and provide a more comprehensive understanding of evolutionary processes across taxa.

### 4.3. Variation in Adaptive Rates Across Species

The rate of adaptive changes varies significantly among microorganisms, animals, and plants, reflecting differences in their life history traits and genetic architectures. Microorganisms, with their short generation times and high mutation rates, exhibit the fastest adaptive responses, often observable within days or weeks. For example, fungi subjected to successive subculturing over just 20 months have been shown to undergo significant degenerative changes, including reduced fruiting body production, metabolic activity, and stress tolerance, likely due to chromosomal instability and genetic variation. In animals, adaptive rates are generally intermediate, with changes manifesting over months or years due to longer lifespans and more complex life cycles. However, some species, such as *Drosophila melanogaster* (fruit flies), exhibit relatively rapid adaptive changes, with phenotypic shifts observable within weeks under controlled laboratory conditions. Despite these exceptions, most animals fall into the intermediate range of adaptive rates.

In plants, adaptive changes typically occur at a slower pace compared with microorganisms and animals, primarily due to their sessile nature, longer generation times, and reliance on epigenetic mechanisms for stress responses. The extended life cycles of many plant species pose significant challenges for studying adaptive changes, as experiments often require years or even decades to observe measurable evolutionary shifts. However, understanding plants’ adaptation is crucial for unraveling the mechanisms of evolutionary processes in sessile organisms. To overcome these limitations, researchers could leverage rapid propagation techniques, such as tissue culture or clonal propagation, to accelerate the generation of plant materials under controlled conditions. These approaches would enable the study of adaptive changes over shorter timeframes, providing insights into the genetic and epigenetic mechanisms driving plant evolution. By adopting methodologies inspired by microbial and animal studies, plant research can bridge the gap in understanding adaptive rates across taxa and contribute to a more comprehensive framework for evolutionary biology.

## 5. Conclusions

Adaptive changes under controlled conditions arise from the complex interplay of environmental factors, genetic and epigenetic mechanisms, and species-specific biological traits. This review systematically examines the processes and genetic foundations of adaptive changes in microorganisms, animals, and plants, revealing that while research on animals and microorganisms is relatively comprehensive, studies on plant adaptation remain limited. The well-established mechanisms and technologies in animal and microbial systems provide a valuable framework for advancing plant adaptation research. Future studies should focus on elucidating the molecular basis of adaptive changes, particularly in understudied taxa such as plants, by leveraging advanced genomic and epigenomic tools to explore the roles of genetic variation, chromosomal instability, and non-coding RNA regulation. Such investigations will not only deepen our understanding of fundamental evolutionary processes but also offer practical applications for optimizing agricultural, biotechnological, and conservation systems. For instance, harnessing adaptive mechanisms in crops could enhance stress tolerance and yield stability, addressing global challenges such as climate change and food security.

## Figures and Tables

**Table 1:** Phenotypic changes and scope of transgenerational cultivation in animals.

Type of Change	Species	Generations	Phenotypes	Scope and Effectivity of Change	References
Reproductive and fertility-related phenotypes	*Drosophila melanogaster*	10	Number of eggs per female per day, mean egg size, starvation tolerance as time to death by starvation, and recovery time after chill coma	Quickly altered	[33]
	*Heterandria formosa*	2	Reproductive rate	Decreased	[34]
			Offspring size	Increased	
	*Callosobruchus maculatus*	2	Sex-specific and hormetic intergenerational and transgenerational effects on longevity and lifetime reproductive success	Great change	[35]
	*Daphnia pulicaria*	2	Fitness traits (delayed maturation, lower reproductive output, and increased clutch interval)	Decreased	[36]
	*Aurelia coerulea*	10	Polyps’ average budding reproduction rate	Decreased	[37]
Physiological and lifespan-related phenotypes	*Caenorhabditis elegans*	3	Starvation resistance and lifespan	Increased	[38]
	Insect model system	80	Fitness and longevity	Increased	[39]
	Freshwater snail *Physa acuta*	2	Shell size and crushing resistance	Increased	[40]
	Freshwater crustacean *Daphnia lumholtzi*	2	Lag phase	Decreased	[41]
Behavioral and life history traits	*Daphnia* *magna*	150	Behavior and life history traits	Rapidly altered	[42]
	Pea aphids	27	The persistence of the winged phenotype	Decreased	[43]
			The refractory phase duration	Increased	
Morphological traits	Freshwater snail (*Potamopyrgus antipodarum*)	3	Shell shape	Slowly altered	[44]

## Data Availability

All datasets generated for this study are included in the manuscript.
